# Bacterial Compatibility in Combined Inoculations Enhances the Growth of Potato Seedlings

**DOI:** 10.1264/jsme2.ME16127

**Published:** 2017-02-04

**Authors:** Christine D. Santiago, Shogo Yagi, Motoaki Ijima, Tomoya Nashimoto, Maki Sawada, Seishi Ikeda, Kenji Asano, Yoshitake Orikasa, Takuji Ohwada

**Affiliations:** 1 United Graduate School of Agricultural Sciences, Iwate University18–8 Ueda-sanchome, Morioka, Iwate 020–8550Japan; 2 Department of Food Science, Obihiro University of Agriculture and Veterinary MedicineInada-cho, Obihiro, Hokkaido 080–8555Japan; 3 Hokkaido Agricultural Research Center, National Agriculture and Food Research Organization9–4 Shinsei-minami, Memuro-cho, Kasai-gun, Hokkaido 082–0081Japan

**Keywords:** plant growth-promoting bacteria, plant tissue localization, combined inoculation, bacterial compatibility

## Abstract

The compatibility of strains is crucial for formulating bioinoculants that promote plant growth. We herein assessed the compatibility of four potential bioinoculants isolated from potato roots and tubers (*Sphingomonas* sp. T168, *Streptomyces* sp. R170, *Streptomyces* sp. R181, and *Methylibium* sp. R182) that were co-inoculated in order to improve plant growth. We screened these strains using biochemical tests, and the results obtained showed that R170 had the highest potential as a bioinoculant, as indicated by its significant ability to produce plant growth-promoting substances, its higher tolerance against NaCl (2%) and AlCl_3_ (0.01%), and growth in a wider range of pH values (5.0–10.0) than the other three strains. Therefore, the compatibility of R170 with other strains was tested in combined inoculations, and the results showed that the co-inoculation of R170 with T168 or R182 synergistically increased plant weight over un-inoculated controls, indicating the compatibility of strains based on the increased production of plant growth promoters such as indole-3-acetic acid (IAA) and siderophores as well as co-localization on roots. However, a parallel test using strain R181, which is the same *Streptomyces* genus as R170, showed incompatibility with T168 and R182, as revealed by weaker plant growth promotion and a lack of co-localization. Collectively, our results suggest that compatibility among bacterial inoculants is important for efficient plant growth promotion, and that R170 has potential as a useful bioinoculant, particularly in combined inoculations that contain compatible bacteria.

High-yield agricultural crop production is desired to sustain global food security. In order to achieve improved crop productivity, plants need to be provided with essential nutrients for growth and disease prevention. Since plants continuously obtain nutrients from the soil, its productivity needs to be maintained through the replenishment of lost nutrients. Therefore, chemical fertilizers containing basic nutrient requirements such as macro-elements (N, P, K, S, Mg, and Ca) and trace elements (Fe, Mn, Bo, Cl, Zn, Cu, and Mo) are being used to ensure plant nourishment. However, the cost of chemical fertilizers is high and prolonged use may have negative impacts.

The effects of chemicals on the environment, including a deterioration in the balance of elements and disrupted populations of natural microflora in the soil (21) in addition to increased concentrations of nitrates in ground water (25), may eventually result in an irreversible impact on human health (32). Moreover, the excessive use of chemicals for crop fertilization may lead to the accumulation of minerals and nutrients that cannot be easily utilized for plant consumption, ultimately resulting in soil pollution and toxicity (21). In contrast, beneficial bacteria are considered to assist in the absorption of insoluble nutrients by plants from the soil (7). Biological inoculants containing novel strains of beneficial bacteria have been developed to support this process. These bacteria have been shown to assist in plant growth promotion by producing phytohormones such as indole-3-acetic acid (IAA) (10, 13) and protective substances including biofilms (23), initiating the formation of siderophores (17), releasing enzymes such as 1-aminocyclopropane-1-carboxylic acid (ACC) deaminase (8), *β*-1,3-glucanase (29), cellulase (20), chitinase (29), protease and lipase (3), and biologically controlling harmful pathogens (9). Some strains have been reported to support plant growth by providing additional tolerance to environmental stressors such as high levels of heavy metals (19), the presence of salt and other chemicals (6), acidic soil (19), and abrupt changes in temperatures (2). In the Tokachi area, Hokkaido, Japan, the growth of plants has been reduced by environmental stresses such as low pH and aluminum toxicity because of volcanic ash soil; however, beneficial bacteria are expected to improve plant productivity (27). Thus, their adaptability in a stressed environment and compatibility with each other need to be considered when selecting the best bioinoculant because single strains may not effectively function as a plant growth promoter if they have to compete with a diverse population of antagonistic strains.

Previous studies reported that the combined inoculation of bacteria promotes plant growth. Amutha *et al.* (1) demonstrated the beneficial effects of co-inoculating different species of *Azospirillum* on the growth of rice; Mahmood *et al.* (12) examined the influence of combined *Rhizobacteria* sp. and *Agrobacteria* sp. on the growth of the banana; and Castillo *et al.* (4) noted the enhanced growth of sunflower seedlings following a co-inoculation with *Achromobacter xylosoxidans* (SF2) and *Bacillus pumilus* (SF3 and SF4). However, the mechanisms by which bacterial combinations promote plant growth have not yet been elucidated in detail.

Someya *et al.* (31) showed that the potato (*Solanum tuberosum* L. cv. Matilda) accommodates a diverse population of plant-associated bacteria, and nineteen representative strains with high affinity for the roots or tubers were examined for their plant growth-promoting abilities (Kenkyuseika, vol. 539. 2015. Tsukuba Office, Agriculture, Forestry and Fisheries Research Council Secretariat, Japan). In the present study, we selected four leading plant growth-promoting bacteria (PGPB) (*Sphingomonas* sp. T168, *Streptomyces* sp. R170, *Streptomyces* sp. R181, and *Methylibium* sp. R182) from these representative strains, and biochemical tests were conducted to identify the strain with the highest potential as a bioinoculant. An environmental stress test was also performed to identify the strain that may potentially provide stress tolerance to a host plant. We then evaluated the compatibility of the best strain with other strains for plant growth-promoting effects concomitant with plant tissue localization. Our results indicate that bacterial compatibility in the combined inoculation is crucial for enhancing plant growth because of the synergistic effects of compatible bacteria that may be attributed to the increased production of plant growth-promoting substances, along with the co-existence of bacteria in the host plant.

## Materials and Methods

### Bacterial strains and medium

Nineteen representative strains with high affinity for potato roots or tubers (cv. Matilda, one of the common varieties in Hokkaido, Japan) isolated previously (31) were studied for their plant growth promotion (Kenkyuseika, vol. 539. 2015. Tsukuba Office, Agriculture, Forestry and Fisheries Research Council Secretariat, Japan), and the four best PGPB strains, namely, *Sphingomonas* sp. T168 (accession number AB730532), *Streptomyces* sp. R170 (AB730341), *Streptomyces* sp. R181 (AB730352), and *Methylibium* sp. R182 (AB730353) were used throughout the experiments performed herein. Strains T168, R170, R181, and R182 belong to AP6, AC4, AC1, and BP12, respectively, of the OTU-group, as reported previously (31). All strains were grown in R2A medium (BD, Sparks, MD, USA).

### Assessment of biochemical and enzyme activities

IAA production was assessed using the Salkowski assay, as reported by Gopalakrishnan *et al.* (9) with the following modifications: single and combined inoculants (1:1 of each strain) were grown in R2A broth containing 2 mM L-tryptophan (precursor of IAA) at 30°C for 72 h, and after centrifugation, the supernatant (400 μL) was poured into Salkowski reagent (composed of 500 μL of 60% HClO_4_, 17 μL of 0.5 M FeCl_3_, and 350 μL of distilled water) (800 μL) and incubated at 30°C for 30 min in a dark place. IAA production was assessed (*n*=3) by optical density at 530 nm using a spectrophotometer (Ultrospec3100pro, GE Healthcare Life Sciences, Buckinghamshire, UK). At the same time, the number of living cells was measured using plate dilution methods with cell pellets left over after removing the supernatant to evaluate IAA contents cell^−1^ (μg 10^8^ colony-forming units [CFU]^−1^).

Siderophore production was evaluated using the Chrome-Azurol S (CAS) agar diffusion assay (26) with some modifications: holes (6 mm in dia.) made on R2A agar containing 10% CAS (22) were filled with a final bacterial suspension of 24-h-old cultures (35 μL); *i.e.*, single inoculants were equally mixed with R2A broth to reach a final volume of 35 μL, and the combined inoculants had equal volumes of each strain in the mixture for a final volume of 35 μL. After an incubation at 30°C for 7 d, the diameters of the halos that formed around the holes containing a bacterial colony were measured. Siderophore production (*n*=3) is expressed as the ratio of the halo diameter (halo dia. minus colony dia.) per colony diameter (30).

Biofilm production was evaluated using the microtiter plate assay (35) with the following modifications: single and combined inoculants (1:1 of each strain) (100 μL) of 2-d-old cultures in R2A broth were transferred into a 96-well polystyrene microtiter plate and incubated at 30°C for 12 h. After loosely associated bacteria were removed, wells were washed with sterilized distilled water, air dried, and then stained with 1% crystal violet solution (150 μL) for 45 min. The wells were washed again with distilled water, destained with 95% ethanol (200 μL), and 100 μL from each well was then transferred to new microtiter plates. Absorbance at OD_595_ was measured (*n*=3) using a microplate reader (iMark Microplate Absorbance Reader, Bio-Rad Laboratories, Tokyo).

ACC deaminase activity was assessed by the production of α-ketobutyrate (AKB) generated through the cleavage of ACC according to the method reported by Penrose and Glick (16), except that cells were grown in R2A broth. At the end of the assay, absorbance at OD_540_ (*n*=3) was measured using a spectrophotometer. Activity was expressed as nmol AKB (mg wet weight of cell)^−1^ h^−1^.

Cellulase, protease, lipase, and chitinase activities were evaluated by the size of the halo diameter that formed from the periphery of the colony at 30°C, 7 d after the bacterial spot inoculation (5 μL) on agar medium (*n*=3). Cellulase activity was estimated according to the method reported by Crabbe *et al.* (5) using yeast-extract salts (YES) agar medium containing 2% carboxymethyl cellulose. Protease and lipase activities were measured according to the method reported by Bhattacharya *et al.* (3) using skim milk agar (3% skim milk and 1.5% agar), and R2A agar supplemented with 1% Tween 20 and 0.01% CaCl_2_·2H_2_O, respectively. Chitinase activity was evaluated according to the method reported by O’Brien and Colwell (14) using R2A agar supplemented with 1% colloidal chitin. Regarding lipase and chitinase, only the presence or absence of their activities was noted because of the indistinct boundaries of halos produced.

*β*-1,3-glucanase activity was assessed according to the method reported by Singh *et al.* (29) using R2A broth supplemented with 1% colloidal chitin. The amounts of reducing sugars were assessed by measuring absorbance at OD_530_ (*n*=3) using a spectrophotometer. One unit of activity was defined as the amount of enzyme that liberated 1 μmol of glucose h^−1^.

### Evaluation of stress tolerance by bacterial strains

A portion of the 24-h pre-culture in R2A broth was added to fresh R2A broth (6 mL) in L-tubes adjusted to different levels of AlCl_3_ (0.0001%–1% [w/v]), NaCl (1.0%–5.0% [w/v]), pH (4.0–10.0), and temperature (10–40°C), and growth was monitored (*n*=3) at OD_660_ by a biophotorecorder (TVS062CA, Advantec Toyo Kaisha, Tokyo). In the aluminum, salt, and pH stress tests, each culture was incubated with shaking at 30 rpm at 30°C for 120 h, and in the temperature stress test, each culture was incubated at a temperature between 10 and 40°C under the same conditions as those used for the shaking and incubation periods described above.

### Bacterial inoculation and evaluation of plant growth promotion

The R170 and R181 strains were both grown in R2A broth at 30°C for 24 h with shaking at 130 rpm. After centrifugation at 10,000 rpm, 4°C, for 5 min, the cell suspension was adjusted to 1×10^8^ CFU mL^−1^ in sterilized distilled water. The T168 and R182 strains were grown on R2A agar medium for 72 h under the same temperature conditions described above. Cells were collected directly from agar medium, and the cell suspension was adjusted to the cell density described above.

In order to evaluate the compatibility of strains when formulating a bioinoculant for plant growth promotion, we focused on the combination of R170, which exhibited the highest potential as a bioinoculant with T168 or R182, with a parallel combination using R181 instead of R170. We directly inoculated single (1 mL) and combined cell suspensions (2 mL; 1 mL from each strain) on potato seeds (*n*=18) derived from the open pollination of cv. Hokkaikogane sown on pots containing approx. 100 g of sterilized seedling-raising culture soil (PotAce N, Katakura & Co-op Agri, Tokyo). The pots were covered with aluminum foil and placed in a growth chamber under light (23.5°C for 14 h) and dark (20.0°C for 10 h) conditions, respectively. The cover was removed upon the germination of seeds, and 30 d after the inoculation, plant growth parameters such as plant weights (mg) and germination rates were measured (*n*=3). Plant dry weight was measured after oven drying at 60°C for 3 d.

### Tissue localization of inoculated strains in plant roots

In order to observe the plant tissue localization of strains T168 and R182, we introduced the *gusA* gene encoding *β*-glucuronidase (GUS) (34) into cells. Regarding strain T168, the plasmid pHRGFPGUS (18), which expresses the *gusA* and *gfp* genes constitutively under the control of a gentamycin resistance gene promoter, was introduced into cells by electroporation using a MicroPulser™ in accordance with its operating instructions (Bio-Rad Laboratories). Regarding strain R182, the plasmid pmTn*5*SS*gusA20* (34), which expresses the *gusA* gene constitutively under the control of an *aph* promoter, was introduced into cells by bi-parental mating according to the method reported by Simon (28). Briefly, *Escherichia coli* S17-1 (donor) and R182 strain (recipient) cells in the exponential phase were mixed together and centrifuged for 3 min. After cell pellets were suspended in 50 μL of 0.85% NaCl, mating was performed on a mixed cellulose ester membrane filter (pore size 0.45 μm, Advantec Toyo Kaisha) placed on R2A agar medium at 30°C for 2 d. Cell suspensions were spread on R2A agar medium containing spectinomycin (50 μg mL^−1^), streptomycin (50 μg mL^−1^), and fosfomycin (50 μg mL^−1^). The T168 and R182 transformants constructed by the introduction of reporter gene(s) were both confirmed to exert similar effects to their parents for plant growth promotion.

Approximately 14-d-old potato seedlings were grown on plant agar (0.3%) containing a 500-fold dilution of HYPONeX^®^ 6-10-5 (HYPONeX Japan, Osaka, Japan) and inoculated with a single or combined inoculant prepared as described above. We identified the tissue localization of the single and combined inoculants in the plant roots by GUS and/or Gram staining. In plants inoculated with GUS-marked strains, bacterial cells were stained by immersing plant samples in a GUS-staining solution (16 mL of 125 mM sodium phosphate; 80 μL of 0.5 M Na_2_EDTA, pH 8.0; 800 μL of 2% X-Gluc [5-bromo-4-chloro-3-indolyl-*β*-D-glucuronic acid] cyclohexylammonium salt; 80 μL of 10% SDS; and 23.6 mL of distilled water) with continuous deaeration in a desiccator connected to a vacuum pump for 30 min, and were then incubated on plants at 30°C for 3 d.

Gram staining was performed according to the instructions of Favor G “*Nissui*” (Nissui Pharmaceutical, Tokyo) with slight modifications. Briefly, plants inoculated with strain R170 or R181 were soaked in Victoria blue solution for 1 min, washed with distilled water, submerged in a destaining reagent for 5 min, and then washed again with distilled water to remove excess staining.

We examined the tissue localization of inoculants in plant roots in 70% glycerol under a light microscope (IX70 Inverted Microscope, Olympus, Tokyo) until 28 d after the inoculation. Photomicrographs were taken using a high-sensitivity CCD camera (VB-7000, Keyence, Osaka, Japan).

### Cross-streak test between co-inoculated strains

Each of the co-inoculated strains was grown in R2A agar medium at 30°C for at least 3 d and then streaked perpendicularly on freshly prepared R2A agar medium; *i.e.*, after the first strain was allowed to grow at 30°C for 3 d, the second strain was streaked at an angle of approx. 90° going outward from the emerged colonies of the first strain. The second colony was incubated at 30°C for another 3 d. We then obtained photographic documentation of the agar plates, including those showing colony lines and inhibition zones that appeared at the intersection of the paired strains.

### Statistical analysis

All experiments were performed with a minimum of three replicates for each treatment. The statistical analysis was performed using SPSS Statistics for Windows v.22.0. Data were either subjected to an analysis of variance (ANOVA) or the Student’s *t*-test. A post-hoc comparison of mean values among treatments was performed using Tukey’s honestly significant difference (HSD) test at the 5% confidence level.

## Results

### Biochemical and enzyme activities

**Fig. 1:**
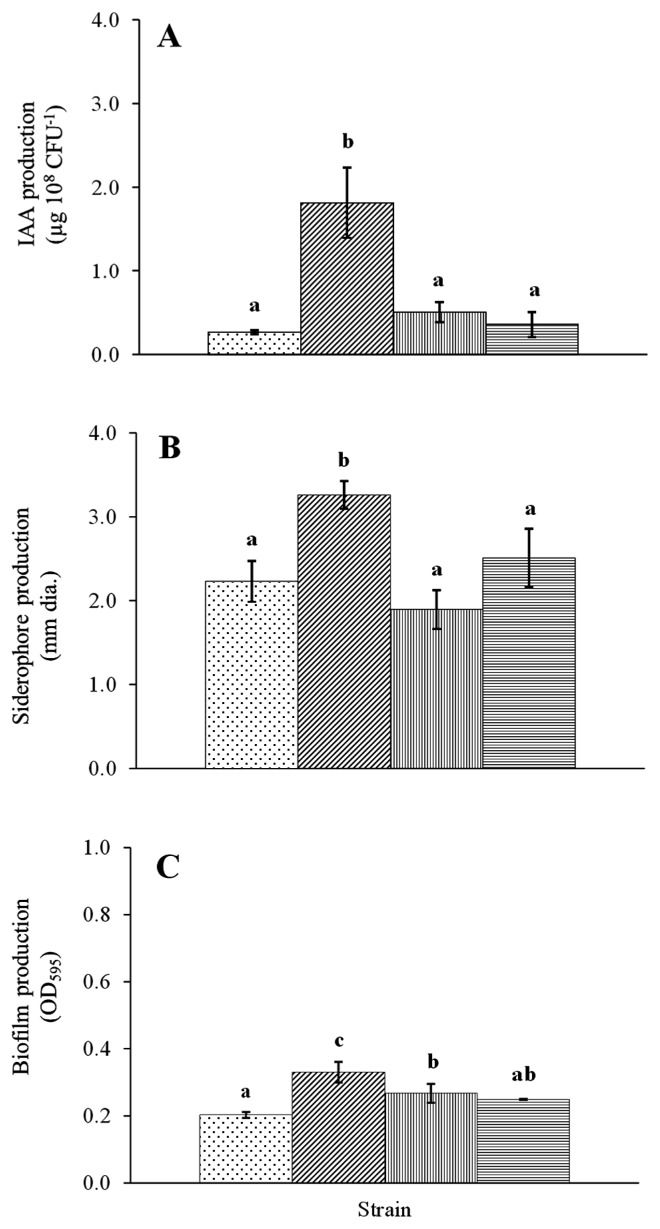
Biochemical activities of four plant growth-promoting bacteria (PGPB) strains. Vertical axes show (A) IAA; (B) siderophore; and (C) biofilm production by each strain. Data are the means±standard deviation (SD) of three replicates. Means in the same parameter (IAA, siderophore, biofilm) with different letters are significantly different from each other at *p*≤0.05. 
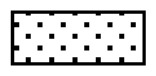
, T168; 
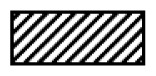
, R170; 
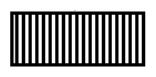
, R181; 
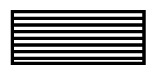
, R182.

We examined the four PGPB strains with high affinities for potato roots or tubers (cv. Matilda) (*Sphingomonas* sp. T168, *Streptomyces* sp. R170, *Streptomyces* sp. R181, and *Methylibium* sp. R182) in order to assess their biochemical and enzyme activities (Fig. 1, Table 1). The results of the biochemical tests showed that even though all four strains produced IAA (*p*=0.000), R170 exhibited the most efficient production with 1.8 μg IAA 10^8^ CFU^−1^ at 72 h (Fig. 1A). The production of siderophores (*p*=0.001) and biofilms (*p*=0.001) was also observed in all four strains; however, the levels achieved were the highest for R170 (Fig. 1B, C).

Regarding enzyme activities, ACC deaminase activity (*p*=0.000) was observed in all four strains and ranged between 421.8 and 690.6 nmol AKB mg^−1^ cell h^−1^, with R170 showing significantly higher activity, followed by R182, R181, and T168 in that order (Table 1). Regarding the production of other enzymes, *β*-1,3-glucanase (*p*=0.004) and cellulase (*p*=0.000) activities were the highest in R182, followed by R170, R181, and T168 in that order. Protease activity (*p*=0.000) was also observed for all strains, except T168, whereas lipase and chitinase activities were only observed for R170 and R182, respectively (Table 1).

### Environmental stress tolerance

**Fig. 2:**
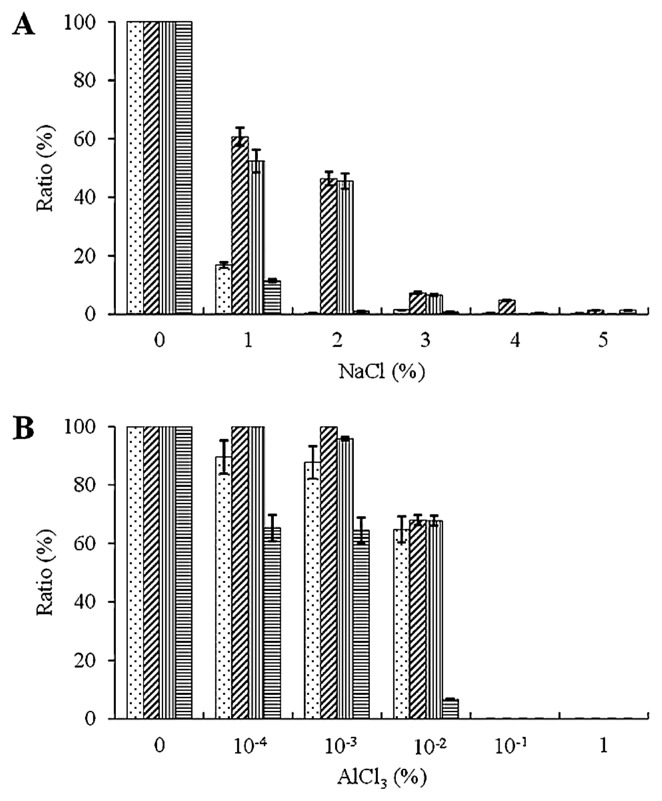
Growth of four PGPB strains at various concentrations of (A) NaCl and (B) AlCl_3_. Vertical axes show the ratio (%) of cell density (OD_660_) (96 h) to that without these compounds at various concentrations of NaCl or AlCl_3_. Data are means±SD of three replicates. The SDs of the means were less than the 0.05 significance level. 
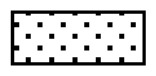
, T168; 
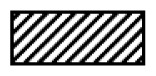
, R170; 
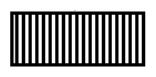
, R181; 
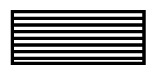
, R182.

Fig. 2 shows the effects of NaCl (*p*≤0.05) and AlCl_3_ (*p*≤0.05) stresses on the growth of the four PGPB strains. Among these strains, R170 showed the highest tolerance to NaCl with approx. 61%, 46%, 7%, and 5% growth over the control at 1%, 2%, 3%, and 4% NaCl, respectively (Fig. 2A). R181 showed similar results with R170 having approx. 52%, 46%, and 7% growth at 1 to 3% NaCl, but a lower tolerance to 4% NaCl (Fig. 2A). T168 and R182 were more sensitive to NaCl stress, showing only 17% and 11% growth even at 1% NaCl, respectively (Fig. 2A).

In terms of AlCl_3_ stress, the growth of all strains was recorded up to 0.01% (Fig. 2B). However, R170 and R181 showed higher tolerance than the other two strains, and the tolerance of R170 was slightly higher than that of R181; at 0.0001% AlCl_3_, R170 and R181 showed similar growth to that of the control, whereas T168 and R182 showed 90% and 65% growth, respectively. At 0.001% AlCl_3_, R170 still showed similar growth to the control, whereas R181, T168, and R182 showed 96%, 88%, and 64% growth, respectively. At 0.01% AlCl_3_, R170, R181, T168, and R182 showed 68%, 67%, 65%, and 7% growth, respectively. Aluminum concentrations higher than 0.1% were lethal for all strains (Fig. 2B).

**Fig. 3:**
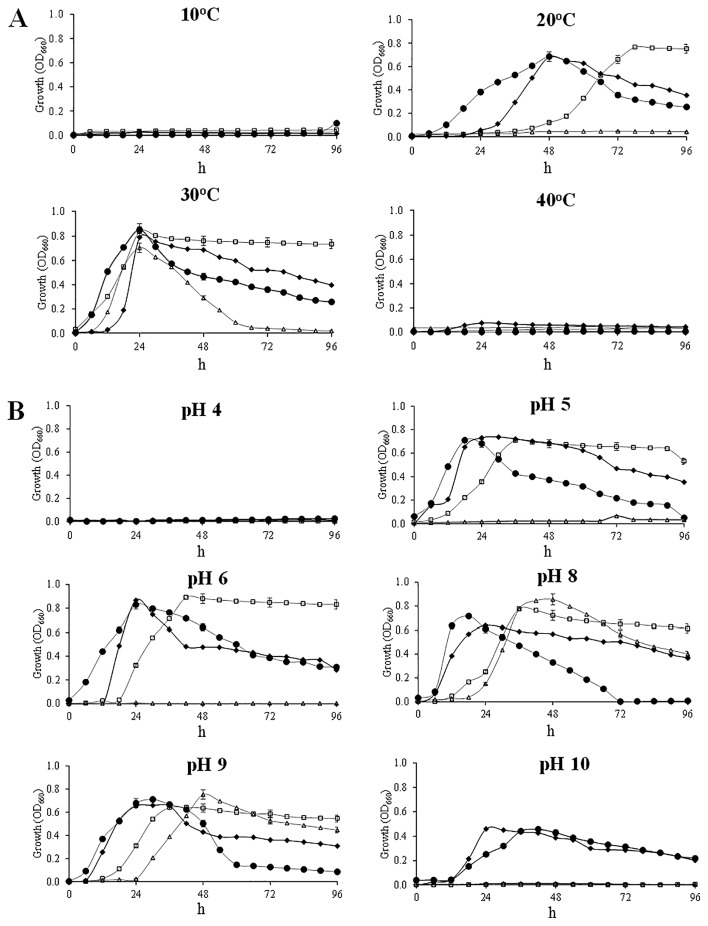
The growth of four PGPB strains under different values for (A) temperature and (B) pH. Growth was monitored at OD_660_ by a biophotorecorder. Data are the means±SD of three replicates. The SDs of the means were less than the 0.05 significance level. □, T168; •, R170; ♦, R181; ▵, R182.

Fig. 3 shows the growth profiles of the four PGPB strains at different ranges of temperature (*p*≤0.05) and pH (*p*≤0.05). The most favorable temperature for all strains was approx. 30°C with a growth peak being observed 24 h after the incubation. At 20°C, the growth of R170, R181, and T168 was delayed with peaks being recorded at 48, 48, and 72 h, respectively, whereas no growth was noted for R182. Among the four strains, R170 started to grow earlier than the other strains. At 10 and 40°C, the growth of all four strains was severely inhibited until at least 96 h after the incubation (Fig. 3A).

Regarding pH, T168, R170, and R181 showed similar growth profiles at pH 5.0 and 6.0, although T168 exhibited a slight delay (Fig. 3B). An alkaline condition (pH 8.0 and 9.0) caused the growth of T168 and R182 to be slightly slower than that of R170 and R181. Strains R170 and R181 survived at high pH (10.0), and the growth of all four strains was inhibited at the low pH of 4.0.

### Effects of combined inoculation on the growth of potato seedlings and germination

R170 was identified as having the highest potential as a bioinoculant based on the results obtained for biochemical and enzyme activities with environmental stress testing. The compatibility of R170 with other strains (T168 and R182) was evaluated for plant growth-promoting ability (Table 2). The results obtained indicated that the combined inoculation of R170 with T168 or R182 significantly improved the growth of potato seedlings over that of the un-inoculated control in terms of fresh (*p*=0.000) and dry weights (*p*=0.000). Accordingly, the increased levels of dry weight (fresh weight) over the control were more than the sum total of those by each strain at approx. 80% (59%) for the combination of R170 with T168, whereas only approx. 65% (31%) was observed for the sum total of each strain. Moreover, an approx. 80% (62%) increase over the control was recorded for the combination of R170 and R182, whereas only approx. 62% (31%) was noted for the sum total of each strain (Table 2).

In contrast, a parallel inoculation test using R181 with T168 or R182 showed no significant difference in plant dry weights from the control. Moreover, the increased levels for both combinations were slightly lower than the sum total of those by each strain; in particular, the levels observed were close to those achieved by the R181 single inoculation. Thus, approx. 25% was recorded for the combination of R181 and T168 or R182, whereas approx. 22% was noted for the R181 single inoculation, based on dry weight over the control (Table 2). Similarly, the co-inoculation of strain T168 with R182, as well as R170 with R181 showed a slight increase in plant dry weights over the control, and lower plant weights than the sum total of each strain (T168 and R182 or R170 and R181), indicating the lack of synergistic effects on plant growth promotion. Regarding the germination rate of potato seeds, no significant effect was observed in the single (*p*=0.136) or combined inoculations (*p*=0.655).

### Additive effects of combined strains on biochemical activities

**Fig. 4:**
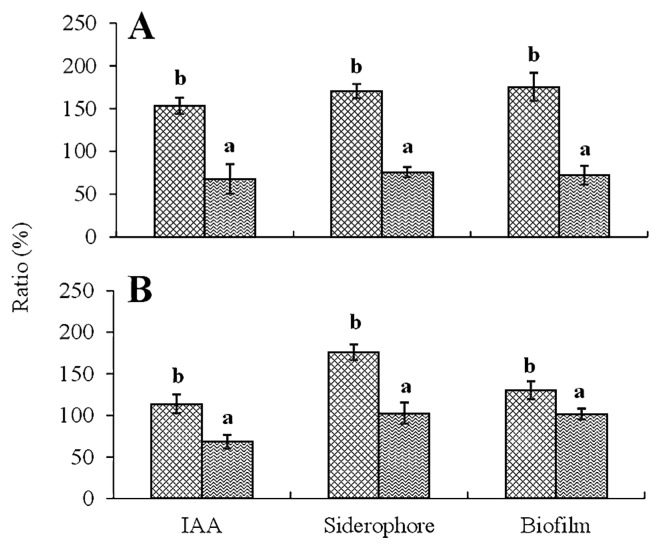
Additive effects of combined strains on the production of IAA, siderophores, and biofilms. Vertical axes show the ratio (%) of IAA, siderophores, and biofilms produced by the combined strains to the sum total of IAA, siderophores, and biofilms produced by each of them. A: Production ratio (%) by the combination of T168 with R170 or R181. B: Production ratio (%) by the combination of R182 with R170 or R181. Data are the means±SD of three replicates. Mean production ratios in the same parameter (IAA, siderophore, and biofilm) with different letters are significantly different from each other at *p*≤0.05. A: 
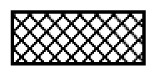
, T168 and R170; 
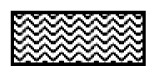
, T168 and R181. B: 
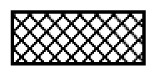
, R182 and R170; 
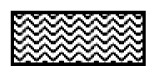
, R182 and R181.

The additive effects of combined strains on biochemical activities are shown in Fig. 4. The results obtained showed that the production of IAA, siderophores, and biofilms were improved by the combination of two compatible strains (R170 and T168 or R182), and these levels increased more than those of the sum total produced by each strain as follows. IAA levels were increased by approx. 53% (*p*=0.002) (R170 and T168) and 13% (*p*=0.005) (R170 and R182), siderophore levels were increased by 70% (*p*=0.000) (R170 and T168) and 76% (*p*=0.001) (R170 and R182), and biofilm levels were increased by 76% (*p*=0.001) (R170 and T168) and 30% (*p*=0.017) (R170 and R182) over the sum total produced by each strain (Fig. 4). However, the parallel test, using R181 with T168 or R182, showed no significant increases in IAA, siderophore, or biofilm production (*p*≤0.05).

### Characteristic localization of inoculated strains in roots of potato seedlings

**Fig. 5:**
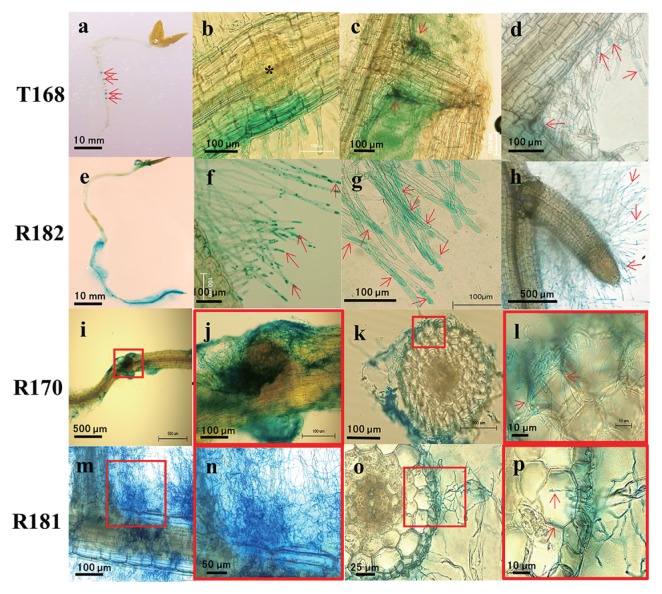
Characteristic localization of inoculated strains in the roots of potato seedlings. a: T168 spots on the roots (at 7 d [7d]). b: Strong infection near the primordium of the lateral root (3d). c: Intense stain at the base of the lateral root (7d). d: Negligible infection at the tips of root hairs (7d). e: Infection of R182 to the entire root surface including root hairs (7d). f: Infection spread from the root hair tips (<1d) (g) to the base (7d). h: Absence at the base of lateral roots (7d). i, j: Massive hyphal growth of R170 surrounding the lateral root (2d) and (k, l) into the inter- and/or intracellular spaces of the main root (cross-section) (7d). m, n: Mass of R181 hyphae surrounding the main and lateral roots (7d) and (o, p) inside the cortical layer of an adventitious root (cross-section) (14d). Major infected regions are shown by red arrows and boxes. Pictures of R170 (i, k) and R181 (m, o) are enlarged in boxes j and l, and n and p, respectively, to show a clearer view of the hyphae.

Fig. 5 shows the characteristic plant tissue localization of the four strains in their initial interaction with the roots of potato seedlings. All strains exhibited efficient colonization as demonstrated in GUS- or Gram-stained plant tissues. Specifically, T168 showed localization at the base of lateral roots (Fig. 5c, d), and R182 was noted on the root hairs and root surfaces (Fig. 5e–h). However, the two species of *Streptomyces*, R170 (Fig. 5i–l) and R181 (Fig. 5m–p) showed random infection and partially covered the plant roots.

T168 appeared to readily localize near the base of root emergence 3 to 7 d before the formation of new lateral roots (Fig. 5b), and appeared as spots on the roots (Fig. 5a). Negligible infection was shown by T168 at the tips of the root hairs (Fig. 5d). However, unlike T168, R182 started to localize near the tips of the root hairs (Fig. 5f) and then spread to the whole root hairs and root surface, as shown with a visible heavy GUS stain (Fig. 5e, g). In addition, R182 was not observed at the base of lateral roots (Fig. 5h). The infection of R170 started to be randomly scattered on the surfaces of the roots (Fig. 5i, j). Endophytic infection was confirmed at 7 d, as shown by the extension of hyphae into the inter- and/or intracellular spaces of the roots (Fig. 5k, l). Similarly, R181 was observed to have an initial random and scattered infection on the surfaces of the roots (Fig. 5m, n). Partial infection into the inter- and/or intracellular spaces of the main root was also observed with R181 (Fig. 5o, p).

**Fig. 6:**
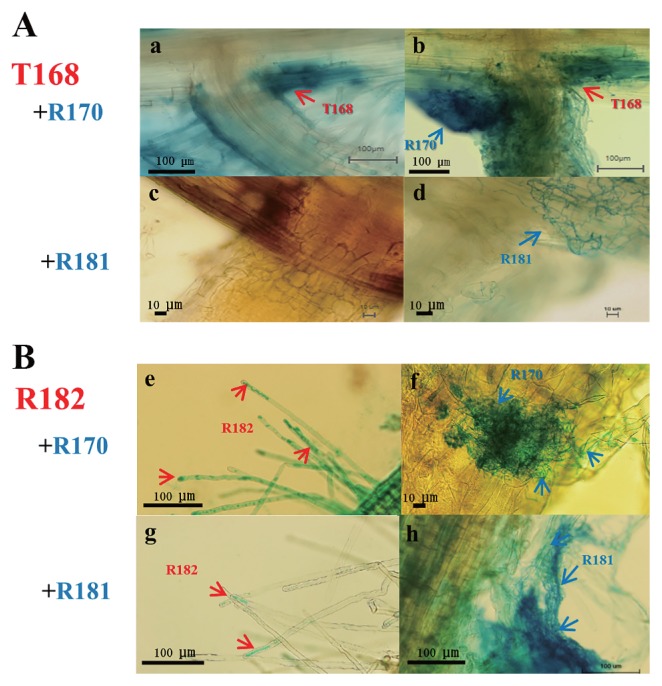
Tissue localization of co-inoculated strains in roots of potato seedlings stained with GUS for T168 and R182; counterstained with Victoria blue (Gram stain) for R170 and R181. A: (a) Localization of T168 at the base of the lateral root in the combined inoculation (T168+R170) that resulted in higher plant growth promotion confirmed by (b) the co-existence of both strains (7 d after the inoculation); and (c) the absence of T168, and localization of R181 at the base of the lateral root (7 d after the inoculation) in the combined inoculation (T168+R181), which showed lower plant growth promotion. B: (e) Localization of R182 that resulted in higher plant growth promotion (R182+R170) confirmed by intense infection to the root hairs by R182, and (f) R170 on the same primary root (7 d after inoculation). g: Localization of R182 in lower plant growth promotion (R182+R181) caused by the weak infection of R182 at the root hairs. h: Localization of R181 that resulted in lower plant growth promotion (R182+R181) revealed by the weak infection of R182 on root hairs.

Fig. 6 shows the tissue localization of two compatible (R170 and T168 or R182) and incompatible (R181 and T168 or R182) strains in the roots of potato seedlings. In the compatible combination of T168 and R170, the colonization of T168 at the base of lateral roots was the same as the T168 single inoculation after GUS staining (Fig. 6a). Using the same seedling, subsequent Gram staining showed that R170 also localized around the base of lateral roots, indicating the ability of each strain to co-exist (Fig. 6b). In the incompatible combination of T168 and R181, a very weak infection of T168 was observed at the base of lateral roots after GUS staining (Fig. 6c), whereas R181 was noted to partially cover the base of lateral roots after Gram staining (Fig. 6d), indicating that each strain did not co-exist because the infection of T168 was inhibited by the presence of R181.

In another compatible combination, *i.e.*, that of R182 and R170, the localization of R182 on the root hairs was observed in the same manner as in the R182 single inoculation after GUS staining (Fig. 6e). Gram staining subsequently showed that R170 also localized on the primary root of the seedling co-inoculated with R182 (Fig. 6f). However, in the incompatible combination of R182 and R181, the infection of R182 to the root hairs was clearly weak, and advanced infection was not observed during cultivation after GUS staining (Fig. 6g). In contrast, R181 demonstrated very aggressive growth that covered the root hairs on which R182 was expected to localize after Gram staining (Fig. 6h).

### Cross-streak test between each strain of compatible and incompatible combinations

**Fig. 7:**
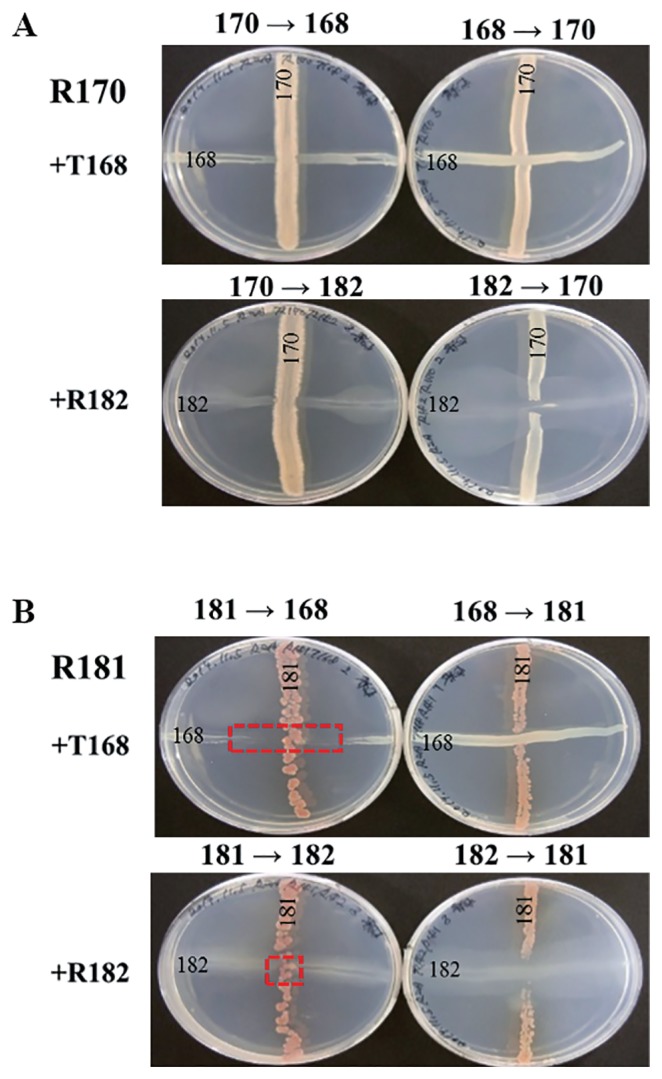
Cross-streak test between co-inoculated strains. Each of the co-inoculated strains was streaked perpendicularly in the order shown using arrows. A: Cross-streak test between combined strains that resulted in higher plant growth promotion (R170 and T168 or R182). No inhibition zone was observed at the intersection between strains. B: Cross-streak test between combined strains that resulted in lower plant growth promotion (R181 and T168 or R182). When R181 was streaked before T168 or R182, an inhibition zone was produced at the intersection, as shown by red boxes.

We examined co-cultures of two compatible (R170 and T168 or R182) and incompatible (R181 and T168 or R182) strains on agar plates (Fig. 7). The compatible combination of T168 and R170, which showed the co-existence of both strains on the plant roots, was reflected by the co-culture of both strains on the same plate with no trace of growth inhibition at the center where the two strains crossed each other (Fig. 7A).

R182 and R170 also showed no growth inhibition between strains even though R170 or R182 was streaked prior to the other strain (Fig. 7A). Clear evidence of growth inhibition was obtained when R181 was streaked first instead of T168 or R182 (Fig. 7B). A sufficient inhibition zone was created by R181, which suppressed the growth of T168, whereas the growth of R182 was slightly inhibited by R181 (Fig. 7B). The growth of R181 was not suppressed when T168 or R182 was streaked on plates prior to R181 (Fig. 7B).

## Discussion

The results of the present study confirmed that the four PGPB strains with high affinity for potato roots or tubers had the ability to produce plant growth-promoting substances as well as tolerance to environmental stress, and also that these strains have potential bioinoculants. The inoculation test with these bacteria, particularly R170, T168, and R182, showed significant increases in the fresh and dry weights of potato seedlings from the control (Table 2), supporting previous findings that these strains have plant growth-promoting abilities (Kenkyuseika, vol. 539. 2015. Tsukuba Office, Agriculture, Forestry and Fisheries Research Council Secretariat, Japan).

In the evaluation of potential strains as bioinoculants, R170 was distinguished as the most suitable candidate due to its ability to produce the highest level of important plant growth-promoting substances (IAA, siderophore, biofilms, and ACC deaminase) among the four PGPB as well as its ability to produce hydrolytic enzymes (*β*-1,3-glucanase, cellulase, protease, and lipase). IAA-, siderophore-, and ACC deaminase-producing bacteria may improve plant growth by promoting root elongation and the proliferation of lateral roots (24), providing bioavailable forms of iron (17), and reducing high levels of ethylene (8), respectively. However, biofilms were noted to indirectly promote plant growth by supporting the establishment of bacterial infections on plants (23, 33). As shown in Table 1, other enzymes such as *β*-1,3-glucanase, cellulase and protease, and lipase have been shown to indirectly influence plant growth (3, 7, 29).

The usefulness of R170 as a potential bioinoculant for plant growth may also be evidenced by its higher tolerance against NaCl and AlCl_3_, and bacterial growth in a wider range of pH values than the other three strains. However, it is not possible to confirm the efficiency of a single-strain bioinoculant unless it is applied in the field, exposed to many external factors (including stress), and interacts with other soil micro-organisms.

Low soil pH, strong aluminum toxicity, and depleted nutrients (*e.g.*, phosphorus), which characterize volcanic ash soil in the Tokachi area, Hokkaido, Japan, have inhibited plant growth and development in the area. Although the application of lime and fertilizers has been reported to overcome these problems, its effectiveness is limited to the soil surface (27). In contrast, bioinoculants containing two or more useful strains that have physiological and biochemical characteristics were also reported to promote plant growth by ensuring the bioavailability of nutrients, while maintaining the balance of soil pH and preventing negative impacts on the environment (15).

Microbial diversity in soil gives rise to a stable ecosystem through the synergistic interactions of compatible microbes, resulting in increased plant productivity (15). In the present study, synergy between the compatible strain R170 with T168 or R182 was evidenced by the higher percentage increase in the plant weight of potato seedlings than the sum total of the plant weight increase using single-strain (T168 and R170, or R182 and R170) inoculants. On the other hand, the absence of synergistic interactions in the incompatible combination (R181 with T168 or R182) was confirmed by the slight increase in plant weight over the sum total of the plant weight increase using single-strain (T168 and R181, or R182 and R181) inoculants. T168 and R182 did not co-exist with R181 on the roots of potato seedlings.

Synergy between strains (R170 with T168 or R182) was also revealed by the improved production of IAA and siderophores, which may have caused the significant increase observed in the weights of potato seedlings. The slight increase in plant weights may be attributed to lower IAA and siderophore production by the incompatible combination (R181 with T168 or R182). The improved production of these plant growth-promoting substances in combined strains may be due to the increased cell number and/or producing activity of either or both bacteria. Further investigations are needed.

The compatibility of strain R170 with T168 or R182 was verified by the lack of inhibition zones at the intersection of two colonies, resulting in co-localization on the roots of potato seedlings. In contrast, the incompatibility of R181 with T168 or R182 on the roots of potato seedlings may be due to growth competition between each bacterium, resulting in the inhibition of T168 at the base of lateral roots and a weakened infection of R182 on the root hairs. Furthermore, the dominance of R181 over T168 or R182 was manifested by the presence of inhibition zones at the intersection of two colonies, which may be related to the production of toxins in the hyphae of R181. This phenomenon has been reported in most *Streptomyces* strains that produce toxins to inhibit the growth of other bacteria and yeasts (11). A thorough assessment based on this assumption for R181 is recommended in further studies.

The performance of compatible strains was tested in a field in Kyushu, Japan, during a double cropping season. The results obtained showed that the co-inoculation of R170 with T168 or R182, with the addition of rice bran, increased the yield of potato over that with no inoculation (data not shown), indicating improved efficiency, particularly in field applications in which environmental stress is high. This result implies that the compatibility of R170 with T168 and R182, in a combined inoculation, promotes plant growth despite stressful conditions.

## Conclusion

The results of the present study suggest that the compatibility of strains in combined inoculations is important for promoting plant growth. Strain R170 was identified as a promising strain (particularly in combination with other compatible strains such as T168 or R182) for the formulation of efficient bioinoculants for potato seedlings, and its use may eventually reduce the utilization of chemical fertilizers.

## Figures and Tables

**Table 1:** Enzyme activities of four PGPB strains

Strain	ACC deaminase (nmol AKB mg^−1^ cell h^−1^)	*β*-1,3-glucanase (U mL^−1^)	Cellulase (mm dia.)	Protease (mm dia.)	Chitinase	Lipase
*Sphingomonas* sp. T168	421.84±9.84^a^	0.15±0.01^a^	0.00±0.00^a^	0.00±0.00^a^	−	−
*Streptomyces* sp. R170	690.60±10.36^d^	0.21±0.01^b^	2.26±0.08^c^	0.18±0.01^b^	−	+
*Streptomyces* sp. R181	494.02±12.54^b^	0.20±0.01^b^	1.58±0.08^b^	1.07±0.05^d^	−	−
*Methylibium* sp. R182	638.68±17.00^c^	0.42±0.01^c^	3.88±0.19^d^	0.48±0.02^c^	+	−

Values are means±SD. A one-way ANOVA was performed to compare the significance of differences between means. Mean values in the same column with common superscript letters are not significantly different from each other (*p*≤0.05) according to Tukey’s HSD test. AKB: α-ketobutyrate. Chitinase and lipase are presented with + or − to indicate the presence or absence of activity, respectively. Regarding *β*-1,3-glucanase activity (U), one unit is defined as the amount of enzyme that liberated one μmol of glucose h^−1^ under the defined conditions.

**Table 2:** Effects of single and combined inoculations on the growth of potato seedlings

Strain	Plant weight (mg)				Germination rate	(%)

	Fresh	(%)	Dry	(%)		
Uninoculated control	226.1±5.4^a^	(100.0)	16.6±0.4^a^	(100.0)	81.5±6.4^a^	(100.0)
**Single inoculation**						
*Sphingomonas* sp. T168	252.7±18.0^bc^	(111.8)	21.8±1.1^b^	(131.3)	92.6±6.4^a^	(113.6)
*Streptomyces* sp. R170	269.8±5.3^c^	(119.3)	22.2±2.0^b^	(133.7)	92.6±3.2^a^	(113.6)
*Streptomyces* sp. R181	239.7±5.2^ab^	(106.0)	20.2±0.1^b^	(121.7)	90.7±6.4^a^	(111.3)
*Methylibium* sp. R182	252.3±5.6^bc^	(111.6)	21.3±0.7^b^	(128.3)	90.7±3.2^a^	(111.3)
**Co-inoculation**						
*Sphingomonas* sp. T168						
+*Streptomyces* sp. R170	359.8±6.6^e^	(159.1)	29.9±1.8^b^	(180.1)	88.9±5.6^a^	(109.1)
+*Streptomyces* sp. R181	261.0±1.3^cd^	(115.4)	20.8±3.4^a^	(125.3)	87.0±3.2^a^	(106.7)
+*Methylibium* sp. R182	263.7±4.8^d^	(116.6)	21.6±1.3^a^	(130.1)	83.5±16.2^a^	(102.5)
*Methylibium* sp. R182						
+*Streptomyces* sp. R170	366.3±1.1^e^	(162.0)	29.9±1.3^b^	(180.1)	92.6±3.2^a^	(113.6)
+*Streptomyces* sp. R181	248.6±28.7^bc^	(110.0)	20.8±3.4^a^	(125.3)	83.3±0.0^a^	(102.2)
*Streptomyces* sp. R170						
+*Streptomyces* sp. R181	239.8±4.3^ab^	(106.1)	18.5±0.1^a^	(111.4)	82.9±11.1^a^	(101.7)

Plant weight and germination rate values are means ± SD calculated from three replicates. A one-way ANOVA was performed to compare the significance of differences among means. Values for single and co-inoculated treatments were separately compared with the uninoculated control. Mean values in the same column with common letters are not significantly different from each other (*p*≤0.05) according to Tukey’s HSD test. The germination rate is shown as the ratio (%) of germinated to ungerminated seeds. The ratio against the uninoculated control is expressed as a percentage in parentheses.
